# Infectious disease aerobiology: miasma incarnate

**DOI:** 10.3389/fcimb.2012.00163

**Published:** 2012-12-19

**Authors:** Chad J. Roy, Doug S. Reed

**Affiliations:** ^1^Tulane National Primate Research Center, Tulane School of MedicineCovington, LA, USA; ^2^Center for Vaccine Research, University of PittsburghPittsburgh, PA, USA

There is an exceedingly small group of microorganisms that are considered pathogenic in humans relative to the microbial biota—with literally just a few bacterial and viral agents that have the potential to cause disease when transmitted in aerosol form. This group of pathogens has adapted to circumvent the rigors of airborne transport to enter the respiratory system and induce infection. The changes that take place in the microbial composition during this process, including expulsion of bioaerosols from an infected host, is a multifaceted process that ultimately effects the underlying functionality of the organism and the induction of disease in the host target. Similarly, the nature of the aerosol source can be determinative of the size distribution of the pathogenic aerosols and thus affect the pattern of initial deposition in the respiratory system and the tissue/cell types most impacted by the pathogen.

The complexity of the mucosal response to infection within the respiratory system is affected not only by the number of infectious particles deposited, but the relative integrity of the microbial constituents contained in the aerosol particles. Conceptualizing and providing a description of the constitutive process of airborne disease transmission have given rise to recent research efforts using both *in vitro* techniques and animal models for the purposes of further defining cellular mechanisms in aerosol-initiated disease pathogenesis. The resulting disease models have shown utility in medical product evaluations targeted to protecting or ameliorating the effects from an infectious mucosal/aerosol challenge. These aerosol disease models have been and continue to be an especially important consideration in biodefense-related research studies.

This special issue brings together 11 articles that are diverse in content but are unified under a central theme of infectious disease aerobiology. This includes focused reviews that survey the current paradigm associated with airborne viral transmission of disease, as well as the benefits of directed alternative (aerosol) treatment of aerosol-acquired disease. The vulnerability of microorganisms in airborne environmental transport is also detailed in 2 of the 11 manuscripts contained in this special issue. Initial host response associated with exposure to airborne pathogens constitutes the majority of the articles included, with special emphasis on experimental infection as well as particle size-dependent disease pathogenesis.

The first article (Milton, 2012) evaluates whether the aerosol modality of infection for smallpox, one of the most infamous infectious plagues of all time, was responsible for the majority of vertical transmission among the human population. A concise survey of what is known historically about vaccination attempts (variolation), data on mucosal infection, the current state of poxviral infection animal models, and epidemiologically-based aerosol transmission studies are all summarized to present conclusions on this route of exposure. This contribution provides an opportunity for the introduction of the term anisotropic infection to describe when the severity of disease is largely contingent on the route of exposure. In the next review article, Hanif and Garcia-Contreras (2012) detail the use of pharmaceutical aerosols for treatment of tuberculosis which is one of only a few truly obligate aerosol diseases. The benefit of therapy directed to the target organ is described with a special emphasis on the historical use of animal models to evaluate targeted delivery of therapies. Much of the data summarized in this review will have applications in designing treatments for other infectious diseases of the lung in addition to tuberculosis.

The next two articles present experimental data associated with microbial survival as it relates to the generation of laboratory-generated infectious bioaerosols for respiratory infection of animals. The first (Faith et al., 2012) presents data that empirically determines the impact of specific changes in microbiological propagation on the overall vulnerability and subsequent infectivity of *Francisella tularensis*. Microbial aerosol efficiencies can be negatively affected by prevailing lower relative humidity as well as the selection of growth media (MHb vs. BHI) used in bacterial propagation. Dabisch et al. (2012) presents data comparing different aerosol sampling devices using *Burkholderia pseudomallei*. Aerosolizing bacteria in tandem with a non-infectious material (polystyrene latex beads) demonstrated differences in collection efficiency which can affect calculation of the delivered dose. These studies demonstrate the importance of microbiological and aerosol characterization when developing experimental animal models for respiratory infection.

The next cluster of articles (Bales et al., 2012; Barnewall et al., 2012a,b; Bowen et al., 2012; Gutting et al., 2012) consider the selection of an animal species and specific traits in the context of aerosol models of disease. The first (Bales et al., 2012) compares lethality and pathogenesis of Rift Valley fever virus (RVFV) after respiratory infection in a variety of inbred rat strains. Stark differences were observed in viral dissemination, disease course, and outcome that were dependent upon the rat strain used. Bowen et al. (2012) compares different methods of respiratory exposure to seasonal (A/PR/8/34) influenza virus in a mouse disease model, contrasting the viral dissemination and lethality estimates. Inhalation of small aerosol particles containing influenza produced a different response than nasal instillation at equivalent doses, which is essential information for the use of this disease model in evaluation of medical countermeasures. The next article (Gutting et al., 2012) contrasts bacterial dissemination among either vaccinated or naïve rabbits aerosol-exposed to *B. anthracis*. The authors found that a pre-existing immune response toward *B. anthracis* spores can disrupt the transport of spores from the lung to the mediastinum which ultimately may affect initiation of infection. Rounding out this group of articles, Barnewall et al. (2012a,b) examines repeated low dose aerosol exposures in rabbits for inhalation anthrax with a focus on the inter-day precision of the aerosol delivery system. Achieving homogeneity in aerosol dosing over multiple days is critically important for efficacy studies.

The next grouping of articles (Barnewall et al., 2012a,b; Gater et al., 2012; Thomas et al., 2012) detail efforts to develop disease models for emerging pathogens. Gater et al. (2012) describe stress associated with the inhalation procedure in aerosol exposed rats. Although higher corticosterone levels were observed as a result of restraint in the nose-only apparatus, no correlative effect was observed in inflammatory response or dissemination of *Yersinia pestis*. The next article (Barnewall et al., 2012a,b) describes a study of monkeypox infection in cynomolgus macaques confirming the inhaled dose in the published literature. Barnewall et al. (2012a,b) also compare the use of gel filters with the “traditional” all-glass impinger to determine delivered dose. Finally, Thomas et al. (2012) describe the role of particle size in infection with *B. pseudomallei*. Bacterial dissemination was altered in mice based upon particle size distribution used in the aerosol exposures. This study represents one of the few that uses particle size modulation to compare disease course using equivalent inhaled doses.

In recent years there has been a noticeable surge of interest in infectious disease aerobiology. In part this has been driven by concerns regarding bioterrorism but also by newly emerging diseases that are primarily aerosol transmitted (such as the SARS coronavirus). The articles in this Research Topic are an excellent cross section of active research and topical reviews which will inform the reader of current technologies, gaps in knowledge, and opportunities for future research.
